# Supplementary figures and images for: PNA6, a Lactosyl Analogue of Angiotensin-(1-7), Reverses Pain Induced in Murine Models of Inflammation, Chemotherapy-Induced Peripheral Neuropathy, and Metastatic Bone Disease
Source: Int J Mol Sci. 2023 Oct 9;24(19):15007. doi: 10.3390/ijms241915007 (PMC10573977; doi:10.3390/ijms241915007)

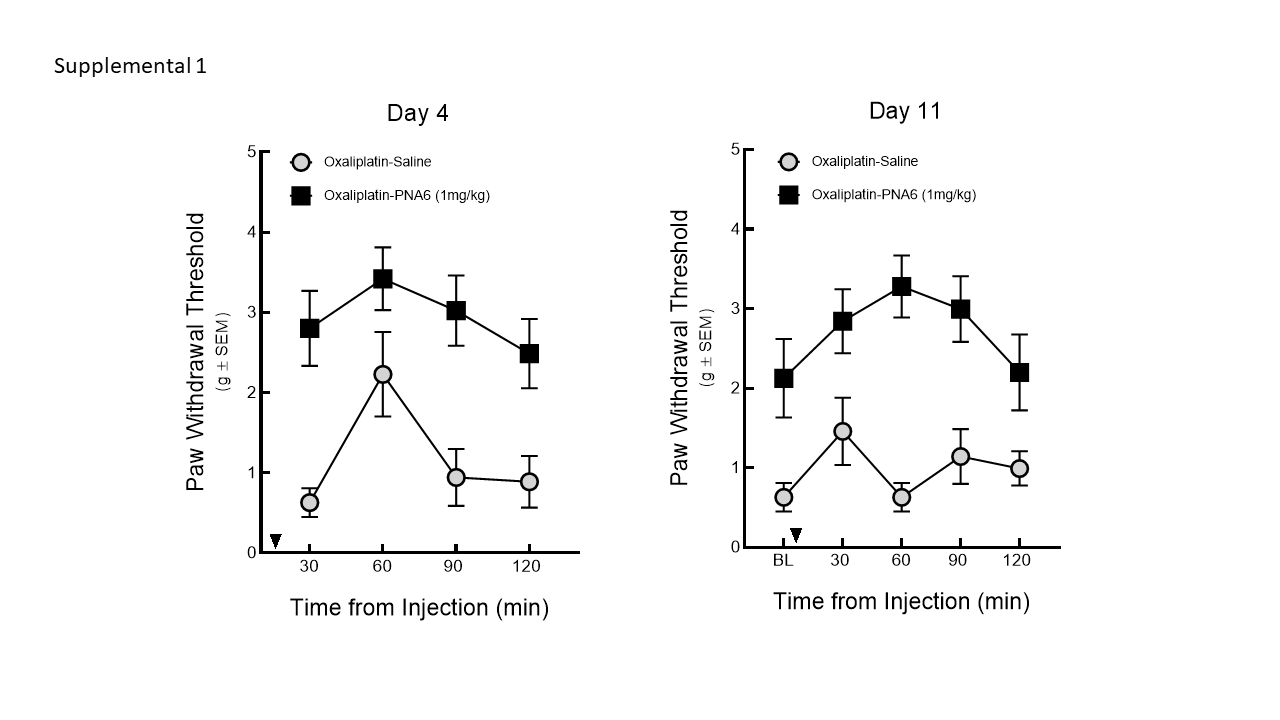

Supplement: Supplementary file 1 [file ijms-24-15007-s001.zip › ijms-2589415-supplementary.TIF]
